# Variation in the incidence of "spontaneous" tumours.

**DOI:** 10.1038/bjc.1979.14

**Published:** 1979-01

**Authors:** R. Schoental


*Br. J. Cancer* (1978) **39**, 101

### Letter to the Editor

### VARIATION IN THE INCIDENCE OF "SPONTANEOUS" TUMOURS

SIR,—In the recent correspondence in your Journal (**38**, 355 (1978)) concerning the striking variation in the incidence of "spontaneous" tumours in BDX rats, Zöller *et al.* suggested that this may be due either to environmental factors or to the development of substrains, as 15–25 generations separated their rats from the original inbred strain.

Among the environmental factors which may influence the incidence of tumours, carcinogenic mycotoxins have to be considered. Fungi, especially those of the *Fusarium* spp., can contaminate cereals harvested during wet and cold weather (Mirocha *et al.* (1976) *Appl. Environ. Microbiol.*, **32**, 553). T-2 toxin (4$\beta$,15-diacetoxy-8$\alpha$-(3-methylbutyryloxy)-12,13-*epoxy*-trichothec-9-en-3$\alpha$-ol), one of the metabolites of several *Fusarium* species, has been found to induce tumours, benign and malignant, in several organs of white rats given intragastric doses of this compound (Schoental *et al.* (1978) *Br. J. Cancer*, **38**, 171).

Occasional presence in the animals' diet of carcinogenic mycotoxins could explain the drastic changes in incidence of "spontaneous" tumours in laboratory animals, transferred to another institute, or occurring in the same laboratory at different times. Recent examples include: a striking decrease of mammary tumours in C3H-$A^{vy}$ mice when the animals were bred in Australia (Sabine *et al.* (1973) *J. Natl Cancer Inst.*, **50**, 1237); a sudden change in the incidence of tumours in a colony of Syrian hamsters, coinciding with the development of vascular lesions (Pour *et al.* (1976) *J. Natl Cancer Inst.*, **56**, 949); the erratic incidence of stomach tumours in *Praomys* (*Mastomys*) *natalensis* (references in Schoental, *Front. Gastrointest. Res.*, in press) and others.

In order to avoid such misleading complications, it is necessary to ensure the absence of mycotoxins in every batch of diet given to laboratory animals. Methods are available to detect not only aflatoxins but also T-2 toxin and certain other *Fusarium* metabolites (Mirocha *et al.*, *ibid.*).

15 September 1978

R. SCHOENTAL
Department of Pathology,
Royal Veterinary College,
University of London,
London, NWI OTU.


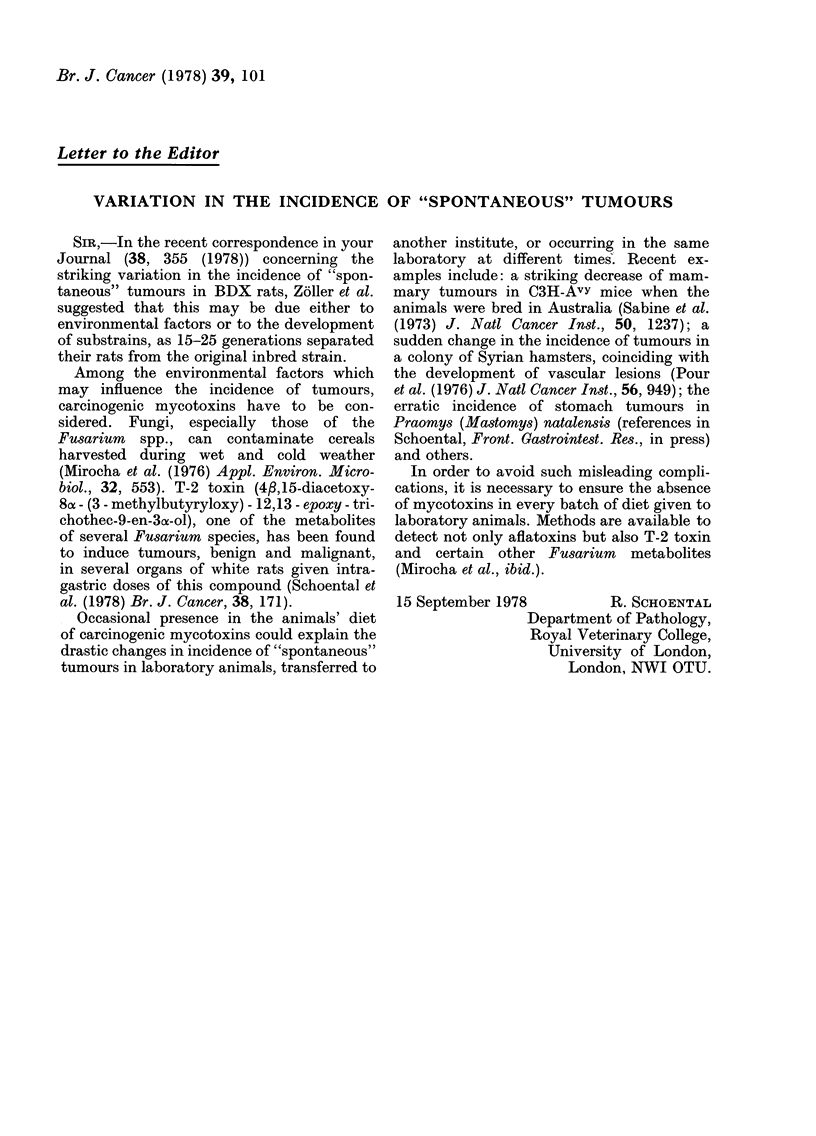


## References

[OCR_00024] Mirocha C. J., Pathre S. V., Schauerhamer B., Christensen C. M. (1976). Natural occurrence of Fusarium toxins in feedstuff.. Appl Environ Microbiol.

